# Use of electronic nicotine delivery systems and cigarette smoking—Add-on vs. displacement dual use

**DOI:** 10.3389/fpubh.2023.1281999

**Published:** 2024-01-04

**Authors:** Knut Kroeger, Vera Helen Buss, Lion Shahab, Martin Storck

**Affiliations:** ^1^Department of Angiology, HELIOS Klinikum Krefeld, Krefeld, Germany; ^2^Department of Behavioural Science and Health, University College London, London, United Kingdom; ^3^Department for Vascular and Thoracic Surgery, Städtisches Klinikum Karlsruhe gGmbH, Karlsruhe, Germany

**Keywords:** smoking cessation, electronic nicotine delivery system, harm reduction, dual use, tobacco

## 1 Introduction

Considering the health harms of tobacco smoking, the primary aim of individual and population-level interventions should always be cigarette cessation [i.e., “tobacco use pattern which involves the cessation of smoking cigarettes,” defined by ADDICTO:0000649 (1)]. However, many smokers struggle to quit, and therefore replacing cigarettes with less harmful electronic nicotine delivery systems (ENDS) such as e-cigarettes can be helpful as a harm reduction strategy. While some people may switch completely, others might prefer dual use of combustible cigarettes and ENDS. Temporary dual use is not an argument against using ENDS as a smoking cessation aid. There is no clear scientific evidence that dual use either increases or decreases harmfulness beyond the level of combustible cigarette use. German guidelines on smoking and tobacco addiction recommend harm reduction through products with low toxicants emission, such as e-cigarettes, for people who smoke combustible cigarettes and are unable to quit smoking or do not want to (2). However, the guidelines also state that dual use leads to much less pronounced reduction in exposure to toxicants compared with completely switching to e-cigarettes. The authors conclude that there is a lack of evidence demonstrating the health impact of dual use due to limited studies in this area, which mostly suffer from methodological problems such as small sample sizes (2).

## 2 Add-on vs. displacement dual use

In contrast to the German guidelines' conclusions on dual use (2), Stokes et al. (3) observed no difference between dual users and those who exclusively smoked cigarettes based on biomarker data (inflammation and oxidative stress) of 7,130 US American adults who used combustible cigarettes, e-cigarettes, both, or none. Further, the researchers found no difference between adults who exclusively vaped and those who did not smoke or vape. Compared with regular cigarette smokers, vapers had significantly lower levels for almost all inflammatory and oxidative stress biomarkers (3). A secondary analysis of a Cochrane systematic review of trials of e-cigarettes for cigarette cessation also demonstrated that the biomarkers are lower when switching to e-cigarettes or dual use compared to combustible cigarette smoking (4). Nevertheless, critics of harm reduction repeatedly portray dual use as dangerous, and sometimes even more so than continued exclusive cigarette smoking (5, 6). By using the term “dual use,” guidelines suggest that there is a generally recognized definition of dual use that forms the basis of these studies and thus for guideline recommendations. However, this is not the case, as we will show below using the studies cited in the German guidelines [(7–10), see Table 1]. Rather, a distinction should be made between add-on use (where cigarette consumption is maintained but topped up with e-cigarettes, e.g., in situations that require temporary abstinence or similar) and displacement dual use (where some cigarettes are actually replaced by e-cigarettes) (11).

**Table 1:** Studies on dual use cited in German guidelines on smoking and tobacco addiction.

**References**	**Study design**	**Findings and comments**
Czoli et al. (7)	Open-label crossover design (*n* = 48) comparing four different scenarios for seven days each: (1) dual use, (2) cigarette use, (3) e-cigarette use, and (4) no product use.	During the entire study, participants used both products to some extent. The period defined as “dual use” fits our description of “add-on use” since participants did not smoke fewer cigarettes than during the week of exclusive cigarette use. During the week of exclusive e-cigarette use, participants reduced their cigarette consumption notably and, hence, had lower levels of carcinogens compared with the cigarette smoking week. Add-on use of e-cigarettes while maintaining similar cigarette consumption did not increase the concentration of measured carcinogens in urine.
Rostron et al. (8)	Cross-sectional study (*n* = 2,700) as part of the Population Assessment of Tobacco and Health (PATH) Study. Participants were categorized into three groups: (1) only cigarette use, (2) dual use of cigarettes and e-cigarettes, and (3) dual use of cigarettes and smokeless tobacco.	The so-called dual users in the second group smoked the same number of cigarettes per day as those who exclusively smoked cigarettes, so the term “add-on use” would have been more appropriate. The add-on use of e-cigarettes to the daily number of smoked cigarettes did not significantly change the urine concentration of a relevant biomarker. This effect was independent of the number of cigarettes smoked.
Shahab et al. (9)	Cross-sectional study (*n* = 181) including: (1) exclusive cigarette smokers, (2) former smokers with long-term (≥6 months) e-cigarette-only, or (3) nicotine replacement therapy-only use, and (4) long-term dual users of combustible cigarettes with e-cigarettes or (5) with nicotine replacement therapy.	The group of dual users smoked, on average, only 2 or 3 fewer cigarettes per day than the group of exclusive smokers, consistent with some minimal displacement. The long-term switch from cigarette smoking to e-cigarette use was associated with significantly lower concentrations of specific carcinogens and toxicants compared with continuous cigarette smoking, while no differences were observed between dual users and exclusive smokers.
Keith et al. (10)	Cross-sectional study (“Cardiovascular Injury due to Tobacco Use Trial,” *n* = 371) including: (1) non-users, (2) exclusively ENDS users, (3) cigarette smokers, or (4) dual users based on their past 30-day consumption.	Smokers and dual users had comparable volatile organic compound metabolite levels. The reported smoking patterns of the two groups did not seem to differ too much in terms of the mean number of daily cigarettes smoked in the past 30 days. Therefore, at least for some study participants, the term add-on use might be more appropriate.

As the studies by Rostron et al. (8), Shahab et al. (9), and Keith et al. (10) were cross-sectional, it is unclear whether the behavior of dual users changed over time. It is possible that more dependent smokers may be more likely to become dual users and so actually reduce their higher cigarette consumption to levels similar to that of less dependent exclusive smokers. Longitudinal comparisons to assess changes in biomarkers have the advantage that researchers can follow up smokers before they start dual using. For example, Pasquereau et al. (12) followed up smokers (exclusive tobacco and dual use of tobacco and e-cigarettes) for 6 months. Those who used both products at baseline were more likely to reduce their cigarette consumption and attempt to quit smoking during the study than those who only smoked cigarettes at baseline. Kasza et al. (13) found that among smokers who were not intending to quit at baseline, those who started using e-cigarettes were more likely to stop smoking within 6 months than those who continued exclusively cigarette smoking. The same effect was observed with nicotine replacement therapy—when offered to smokers, even if they did not intend to quit, they were more likely to make a quit attempt than when not offered nicotine replacement therapy (14).

Using data from the Population Assessment of Tobacco and Health (PATH) Study conducted in the US between 2013 and 2014, Goniewicz et al. (15) observed two distinct usage groups among 792 dual users. One group smoked cigarettes and used e-cigarettes daily. This group could be labeled as add-on users. Another group used e-cigarettes daily but only smoked cigarettes on some days, so could be described as displacement dual users. The former group had significantly higher biomarker concentrations compared with the latter group. The authors concluded that the frequency of cigarette use among those consuming both products was positively correlated with nicotine and toxicant exposure (15). A study funded by Juul labs (an e-cigarette company) using the same PATH Study, but data collected in 2018/19, compared dual users who smoked <10 cigarettes per day (“displacement dual users”) to those who smoked at least 10 cigarettes per day (“add-on users”) (16). Toxicant levels of displacement dual users were lower than those of add-on users, while the levels of add-on users were comparable to exclusive cigarette smokers (16).

ENDS use is associated with a significant reduction in toxicants compared with the consumption of combustible cigarettes. The WHO, known to be rather critical of e-cigarettes, stated in its report on electronic nicotine and non-nicotine delivery systems (EN&NNDS): “There is conclusive evidence that: completely substituting EN&NNDS for combustible tobacco cigarettes reduces users' exposure to numerous toxicants and carcinogens present in combustible tobacco cigarettes; …” (17). The International Agency for Research on Cancer, which forms part of the WHO, states on their website (18): “E-cigarettes have the potential to reduce the enormous burden of disease and death caused by tobacco smoking if most smokers switch to e-cigarettes and public health concerns are properly addressed.” Despite this encouraging assessment, many consider the simultaneous consumption of combustion cigarettes and ENDS as harmful, and the risk of so-called dual use is cited as a strong argument against recommending ENDS use (5, 6). As stated above, the term dual use is not generally well-defined and negative effects of dual use beyond those of exclusive cigarette smoking have not been scientifically substantiated. One can speak of dual use in a completely neutral way when two products are used side by side. However, this is not suitable for scientific evaluation. It is important to distinguish between smokers who have not changed their cigarette smoking pattern but who additionally started using ENDS and those who replaced some of their combustible cigarette consumption through ENDS use. The former could be defined as “add-on use” and the latter as “displacement dual use.”

Add-on use, commonly associated with higher nicotine dependence (19, 20), is not recommended as it does not reduce the level of toxicants inhaled. In contrast, displacement dual use reduces the inhaled concentration of toxicants compared with obtaining the same amount of nicotine by smoking cigarettes exclusively (3, 4, 7–10). The idea of harm reduction in the context of smoking means that people should reduce their cigarette consumption as much as possible by switching to alternatives that contain less harmful toxicants. The publications summarized above have demonstrated that add-on use is not generally associated with an increased concentration of biomarkers (3, 4, 7–10). The measured values correlated with the number of combustible cigarettes consumed. With displacement dual use, on the other hand, the concentration of carcinogens in the urine decreased in line with the decrease in the number of combustible cigarettes smoked, suggesting that ENDS use did not measurably contribute to additional toxicant intake.

## 3 Displacement dual use as a cigarette cessation aid

The European Union and its member states have been trying for years to curb the consumption of tobacco and related products through different measures, including regulations, restrictions on advertising and sponsorship, smoke-free zones, and anti-smoking campaigns. The European Commission regularly conducts opinion polls to gauge Europeans' attitudes toward tobacco-related issues. These polls showed that e-cigarettes and heated tobacco products did not contribute to smoking uptake. A US study (21) using data from the Tobacco Use Supplement to Current Population Surveys and the National Health Interview Survey found that from 2014/2015 to 2018/2019, exclusive ENDS use increased while exclusive cigarette and dual use of ENDS and cigarettes decreased [in the US, dual use primarily fits our definition of add-on use (22, 23)]. In agreement with studies (12, 13) cited above, a 24-month study on the consumption of tobacco and e-cigarettes among young adult binge drinkers showed that dual use is often a transitional phase between cigarette smoking and cessation (24). The latent transition analysis revealed four distinct user patterns among young adults from the US and Canada: (1) exclusive e-cigarette use, (2) dual use, (3) exclusively combustible cigarette smoking, and (4) non-use. Most of the dual users switched to complete abstinence or to the exclusive consumption of e-cigarettes. For smokers who used only combustible cigarettes, the most common transition was abstinence, followed by those who remained in the group of combustible cigarette smoking. After 24 months, 63% of exclusive e-cigarette users transitioned to abstinence, 37% continued to use e-cigarettes, and none transitioned to dual or combustible cigarette use (24).

The German DEBRA study showed that e-cigarette use was associated with higher odds of successful quitting than nicotine replacement therapy use or no aid (25). A study from New Zealand assessed smoking and vaping patterns in people who smoked cigarettes but were not currently using ENDS or were using them less than once a week, not currently attempted to quit, and had never tried to quit through using ENDS for 30 days or more (26). Participants received an ENDS device at the beginning of the study and were asked to report their use over 20 weeks. Most participants reported different consumption levels of combustible cigarettes and ENDS throughout the study period, which also included phases of dual use. The authors concluded that the considerable diversity in alternate use observed within and between study participants suggests that the high variability is typical rather than exceptional. The transition from smoking to ENDS use may involve significant periods of dual use that are likely to be dynamic and may span several months (26).

In qualitative interviews, Notley et al. (27) found that some former smokers started using e-cigarettes without attempting to quit combustible cigarette smoking but slowly transitioned by replacing some of their cigarettes through e-cigarette use, and eventually found more pleasure in e-cigarettes than in combustible cigarettes. Because e-cigarette use, unlike other nicotine replacement products, can substitute psychological, psychosocial, and social aspects of combustible cigarette smoking, it may be more suitable to help some smokers quit cigarettes than other nicotine replacement products. In addition, e-cigarettes offer unique features for smoking relapse prevention (27, 28).

## 4 Conclusions

Unfortunately, there is no recognized definition of dual use in the scientific literature that differentiates between what we term add-on and displacement use dual use. The studies on the topic of dual use listed in the German guidelines on smoking and tobacco dependence illustrate this dilemma clearly. In most of these studies, what is referred to as dual use likely represents add-on use. At the same time, however, these studies also show that even add-on use, regardless of the form in which it is practiced, does not lead to higher levels of toxicant exposure for the consumer than consumption of combustible cigarettes alone. Dual use and add-on use are not the goals of cigarette cessation strategies. The primary goal is the complete cessation of cigarettes. From a health perspective, people would ideally quit all nicotine-containing products. However, for those who cannot achieve this, a full switch to ENDS makes sense, and temporary dual use is not a good argument against using ENDS as an aid to achieve abstinence from cigarette smoking, especially if it leads to later cessation of all nicotine-containing products. There is no scientific evidence that dual use is more harmful than combustible cigarette use if the number of cigarettes smoked remains the same. Therefore, we suggest that the adoption of agreed standards would help to evaluate the consequences of add-on and displacement dual use, respectively. A clearer differentiation would not just be of scientific value but could guide decision-making in clinical practice. Temporary displacement dual use should be evaluated differently than permanent displacement dual use or even add-on dual use. These dual users likely require a different approach to successfully achieve cigarette cessation. If research continues to show that displacement dual use reduces exposure to harmful toxicants compared to exclusive cigarette smoking and potentially increases chances of quit success, it should be recommended by guidelines as a harm reduction tool. After all, the aim of interventions should be to reduce the harm, with abstinence as an ultimate ideal but not a requirement.

## Author contributions

KK: Conceptualization, Writing—original draft. VB: Writing—review & editing. LS: Writing—review & editing. MS: Writing—review & editing.
